# Nutritional Modulation of Oxidative Stress and Metabolic Resilience in Aquaculture

**DOI:** 10.3390/antiox14121515

**Published:** 2025-12-18

**Authors:** Marta Monteiro, Luisa M. P. Valente

**Affiliations:** 1 CIIMAR/CIMAR LA, Centro Interdisciplinar de Investigação Marinha e Ambiental, Universidade do Porto, Av. General Norton de Matos S/N, 4450-208 Matosinhos, Portugal; 2 Instituto de Ciências Biomédicas de Abel Salazar, Universidade do Porto, Rua de Jorge Viterbo Ferreira, 228, 4050-313 Porto, Portugal

Aquaculture continues to expand rapidly, increasing the need for nutritional approaches that mitigate oxidative stress, support metabolic homeostasis, and enhance resilience to environmental and dietary challenges. Oxidative imbalance is a major driver of cellular dysfunction, immune impairment, and reduced performance in farmed species, primarily through excessive production of reactive oxygen species (ROS) and subsequent redox disruption. With the withdrawal of ethoxyquin from commercial aquafeeds and growing restrictions on synthetic antioxidants, there is heightened interest in alternative strategies capable of maintaining oxidative stability in both feeds and fish. Importantly, recent research shows that effective redox management in aquaculture relies not only on the inclusion of direct antioxidant compounds, but also on a broader range of nutritional interventions—such as metabolic regulators, emulsifiers, probiotics, carotenoids, and phytogenic extracts—that can enhance endogenous antioxidant responses by modulating lipid metabolism, cellular stress pathways, immune signaling, and mitochondrial function. Within this context, the contributions in this Special Issue illustrate diverse and innovative nutritional approaches to sustaining redox balance and physiological robustness in aquatic farmed species.

A consistent theme across the contributions is the modulation of hepatic lipid metabolism, cellular stress pathways, and redox balance under metabolically demanding conditions. In largemouth bass (*Micropterus salmoides*), dietary inclusion of rosiglitazone, a synthetic PPAR-γ (peroxisome proliferator-activated receptor gamma) agonist, effectively alleviated high-fat-diet-induced dyslipidemia, hepatic steatosis, and oxidative stress while activating Nrf2 (Nuclear factor erythroid 2-related factor 2)-linked antioxidant defenses and reducing inflammatory markers, after 8 weeks of feeding (Contribution 1). These improvements were associated with enhanced resilience to acute ammonia stress, highlighting the interplay between metabolic regulation, antioxidant capacity, and environmental stress tolerance. In *Litopenaeus vannamei*, the dietary replacement of soybean lecithin with varying levels of soybean lysolecithin (0%, 0.1%, 0.5%, 1%, 1.5%, and 2%) exerted clear dose-dependent effects, with low inclusion (0.1%) improving digestibility, hepatopancreas structure, and endoplasmic reticulum stress markers, while higher levels (>1.5%) promoted lipid accumulation and oxidative susceptibility when compared to a positive control including 2% soy lecithin (Contribution 2). Together, these two studies underscore the relevance of nutritional strategies to improve lipid utilization and maintain redox homeostasis in aquafeeds.

Sustainable ingredients enriched with bioactive antioxidants also feature prominently in this Special Issue. Antache et al. (Contribution 3) showed that insect meals and phytogenic compounds can beneficially modulate oxidation, inflammation, and metabolic regulation in aquatic species. Diets incorporating cricket meal (*Acheta domesticus*) with turmeric (*Curcuma longa*) or beetroot (*Beta vulgaris*) improved growth, reduced oxidative damage, enhanced metabolic efficiency, and modulated hematological parameters in koi carp (*Cyprinus carpio* var. *koi*) (Contribution 3). Furthermore, a comprehensive meta-analysis on astaxanthin (Contribution 4) reflected its recognized potency as a natural antioxidant in aquafeeds. Across 64 studies and 33 species, astaxanthin supplementation consistently improved growth, feed efficiency, antioxidant enzyme activity, immunity, and survival, while also revealing species- and habitat-specific response patterns. A specific example is that the inclusion of astaxanthin in diets for juvenile swimming crabs (*Portunus trituberculatus*) revealed specific metabolic and antioxidant responses (Contribution 5). Moderate supplementation enhanced coloration, antioxidant biomarkers, and lipid metabolism, whereas excessive inclusion led to marked shifts in immune-related gene expression. These studies highlight that astaxanthin can influence innate immune pathways in a non-linear manner, underscoring the need for species- and dose-specific optimization when administering antioxidant compounds.

Agri-food by-products such as pineapple peel and stem flours can be upcycled into aquafeeds, improving feed preservation and modulating the stress physiology of European seabass (*Dicentrarchus labrax*) while promoting a circular economy framework. According to Pereira et al. (Contribution 6), both flours exhibited strong antioxidant activity, before and after storage, and improved feed stability, but they did not enhance stress resistance during an air exposure challenge. Overall, the evidence highlights that antioxidant-enriched sustainable ingredients can confer substantial oxidative benefits to diet stability; however, these effects do not necessarily translate into improved physiological resilience, and their functional outcomes cannot be predicted solely from in vitro antioxidant capacity, requiring confirmation through targeted in vivo testing.

Finally, novel approaches are emerging to enhance antioxidant properties and vitamin C-related effects. A recombinant *Bacillus subtilis* expressing the GULO gene was tested in Nile tilapia to enhance endogenous vitamin C synthesis (Contribution 7). This strategy improved growth, antioxidant status, vitamin C availability, and immune responses, illustrating the potential of gut microbiota modulation to influence host redox balance and stress resilience. However, it is important to note that the application of transgenic or genetically modified microorganisms in commercial aquaculture remains subject to strict regulatory oversight in many regions, namely in Europe where regulations impose stringent authorization, traceability, and environmental risk requirements (EU Regulation (EC) No 1829/2003 on genetically modified food and feed, Directive 2001/18/EC governing the deliberate release of GMOs into the environment, and Directive 2009/41/EC on the contained use of genetically modified microorganisms). Thus, while the biological potential of these recombinant probiotics is clear, their translation into commercial practice will depend on future regulatory developments.

This Special Issue collectively demonstrates that antioxidant nutrition in aquaculture is no longer limited to supplementing direct radical-scavenging compounds. Instead, it encompasses a broader systems-oriented strategy in which metabolic regulators, emulsifiers, carotenoids, phytogenics, and microbiota-targeted probiotics, alone or in combination, modulate lipid metabolism, stress responses, immune signaling, and mitochondrial function to maintain redox homeostasis. Across the studies, a consistent pattern emerges: effective oxidative management depends on precision approaches—appropriate dosing, species- and life stage-specific tailoring, and careful alignment between in vitro antioxidant capacity and in vivo physiological outcomes. Together, these findings advance our understanding of how targeted nutritional modulation can reinforce redox resilience and guide the development of feeds aligned with species-specific physiological requirements.

## Data Availability

Data is contained within the article.

